# Epidemiology and Diversity of Rickettsiales Bacteria in Humans and Animals in Jiangsu and Jiangxi provinces, China

**DOI:** 10.1038/s41598-019-49059-3

**Published:** 2019-09-11

**Authors:** Miao Lu, Feng Li, Yong Liao, Jin-Jin Shen, Jian-Min Xu, Yin-Zhong Chen, Jian-Hua Li, Edward C. Holmes, Yong-Zhen Zhang

**Affiliations:** 1 State Key Laboratory for Infectious Disease Prevention and Control, Collaborative Innovation Center for Diagnosis and Treatment of Infectious Diseases, National Institute for Communicable Disease Control and Prevention, Chinese Center for Disease Control and Prevention, Changping, Beijing China; 2 Yancheng Center for Disease Control and Prevention, Yancheng, Jiangsu province China; 3 Ganzhou Center for Disease Control and Prevention, Ganzhou, Jiangxi province China; 4 Jiangxi Center for Disease Control and Prevention, Nanchang, Jiangxi province China; 5 Marie Bashir Institute for Infectious Diseases and Biosecurity, Charles Perkins Centre, School of Life and Environmental Sciences and Sydney Medical School, The University of Sydney, Sydney, NSW 2006 Australia; 6 Shanghai Public Health Clinical Center & Institutes of Biomedical Sciences, Fudan University, Shanghai, China

**Keywords:** Infectious-disease epidemiology, Infectious-disease diagnostics, Bacterial infection

## Abstract

Diseases caused by Rickettsiales bacteria are a global public health problem. To better understand the diversity and origins of Rickettsiales infection in humans and animals, we sampled 134 febrile patients, 173 rodents and 43 shrews, as well as 358 ticks, from two cities in Jiangsu and Jiangxi provinces, China. Our data revealed a relatively high prevalence of scrub typhus cases in both localities. In addition, both serological tests and genetic analysis identified three patients infected with *Anaplasma bovis*, *Rickettsia monacensis*, and *Orientia tsutsugamushi* bacteria. Molecular epidemiological investigation revealed the co-circulation of multiple species of Rickettsiales bacteria in small mammals and ticks in both provinces, potentially including novel bacterial species. In sum, these data demonstrate the ongoing importance of Rickettsiales infection in China and highlight the need for the regular surveillance of local arthropods, mammals and humans.

## Introduction

Rickettsiales bacteria are obligate intracellular parasites of eukaryotes and responsible for a wide range of important human diseases including anaplasmosis, ehrlichiosis, rickettsioses, and scrub typhus^1^. Importantly, rickettsial diseases have not been effectively controlled worldwide, and scrub typhus remains a major public health concern in most countries where it is endemic^2^. In both developed and developing countries the incidence of human monocytotropic ehrlichiosis (HME) and human granulocytic anaplasmosis (HGA) have increased steadily since their discovery in the 1980s and 1990s, respectively^3–6^. In addition, the application of molecular diagnostic methods in recent decades has resulted in a continual increase in the identification of novel rickettsial bacteria from various samples, as well as their associated diseases^2,7–9^, and bacteria previously considered non-pathogenic are now commonly associated with human diseases^2,8–10^.

In China, the Rickettsiales *Orientia tsutsugamushi*, *Rickettsia prowazekii* and *R*. *typhi* have been important causes of past human morbidity and mortality^2,11,12^ and at least 5,000 human cases (range 5,041~23,540) were recorded each year over the last decade. Of these, scrub typhus is particularly important and has a wide geographic distribution^13^. Although the disease was originally considered to mainly occur in southern China^14^, cases of scrub typhus are now often reported in the northern China^15,16^, reflecting the northward spread of the disease. Human cases of HGA and HME have also been frequently reported since their discovery in Anhui province (in 2006) and Inner Mongolia (in 1999), respectively^17,18^. However, the true number of human infections caused by these rickettsial agents is uncertain due to a lack of commercial diagnostic kits and limited surveillance at both local and national levels. Finally, multiple species of Rickettsiales bacteria associated with human disease have recently been identified throughout China^12^, again emphasizing that rickettsial disease is a major public health problem in this country.

Ticks and rodents play important roles in the transmission of Rickettsiales bacteria in animals and from ticks to humans^19,20^, and recent surveys have revealed a remarkable diversity, prevalence, and geographic distribution of Rickettsiales bacteria in both ticks and rodents^2,11,12,20^. China is home to a great diversity of ticks, with those identified to date being classified into at least 117 species (belonging to 10 genera of 2 families) and with a nationwide distribution^21,22^. The diversity of rodent species in China is also striking, with 171 recorded species^23^.

To better understand the diversity and origins of rickettsial infection in humans in China, particularly their relationship to those co-circulating bacteria present in small mammals and ticks, we collected blood from patients presenting with fever, and concurrently sampled ticks, rodents and shrews from Ganzhou city (Jiangxi province) and Yancheng city (Jiangsu province). Our results revealed that human cases were due to infection by *Rickettsia monacensis*, *Anaplasma bovis*, and *O*. *tsutsugamushi*, and that the local ecology of both regions was characterized by the co-circulation of a diverse range of Rickettsiales bacteria in small mammals.

## Results

### Occurrence of rickettsial disease in Jiangsu and Jiangxi provinces, China

**Figure 1:**
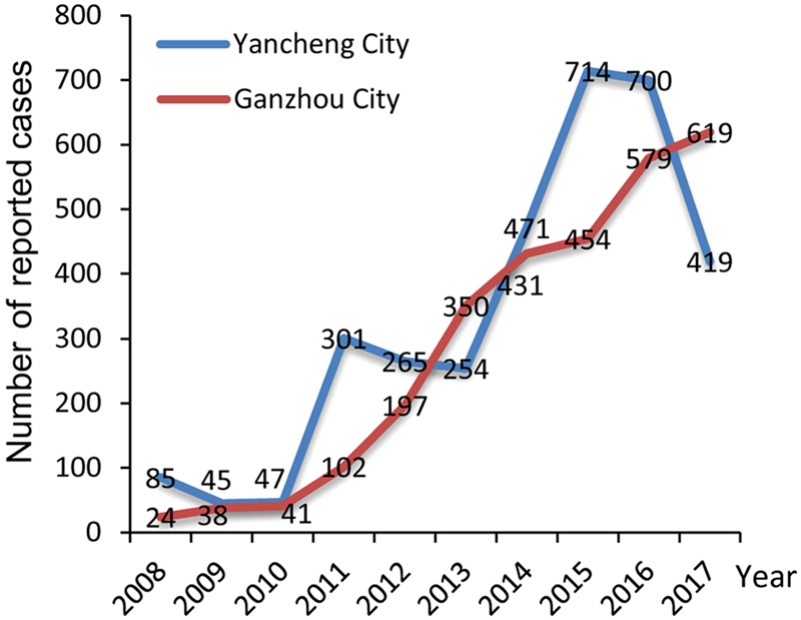
Annual number of cases of scrub typhus in Yancheng and Ganzhou cities, China, 2008–2017.

Rickettsiales diseases have been reported in both Jiangsu and Jiangxi provinces since the 1980s. At least two major epidemics of scrub typhus have occurred in Yancheng city, Jiangsu province. The first epidemics occurred in 1986, with 264 registered clinically diagnosed cases. Fortunately, the number of cases then declined and was relatively stable for the subsequent 20 years (1986–2005). However, the true number of scrub typhus cases is likely higher than recorded because of a combination of inadequate testing and suboptimal reporting. A second epidemic of scrub typhus occurred in 2006, with 149 reported cases. Since then, some 3301 scrub typhus cases have been recorded over the last decade in Yancheng city (Fig. 1), reflecting the high prevalence of *O*. *tsutsugamushi* in animals in Yancheng. In the case of Ganzhou city (Jiangxi province), a total of 2835 clinically diagnosed scrub typhus cases were recorded between 2008–2017, with an annual increase in the number of cases since 2010 (Fig. 1). Due to a lack of commercial diagnostic kits, clinical cases caused by other Rickettsiales bacteria were not recorded.

### Clinical features of Rickettsiales infection

**Figure 2:**
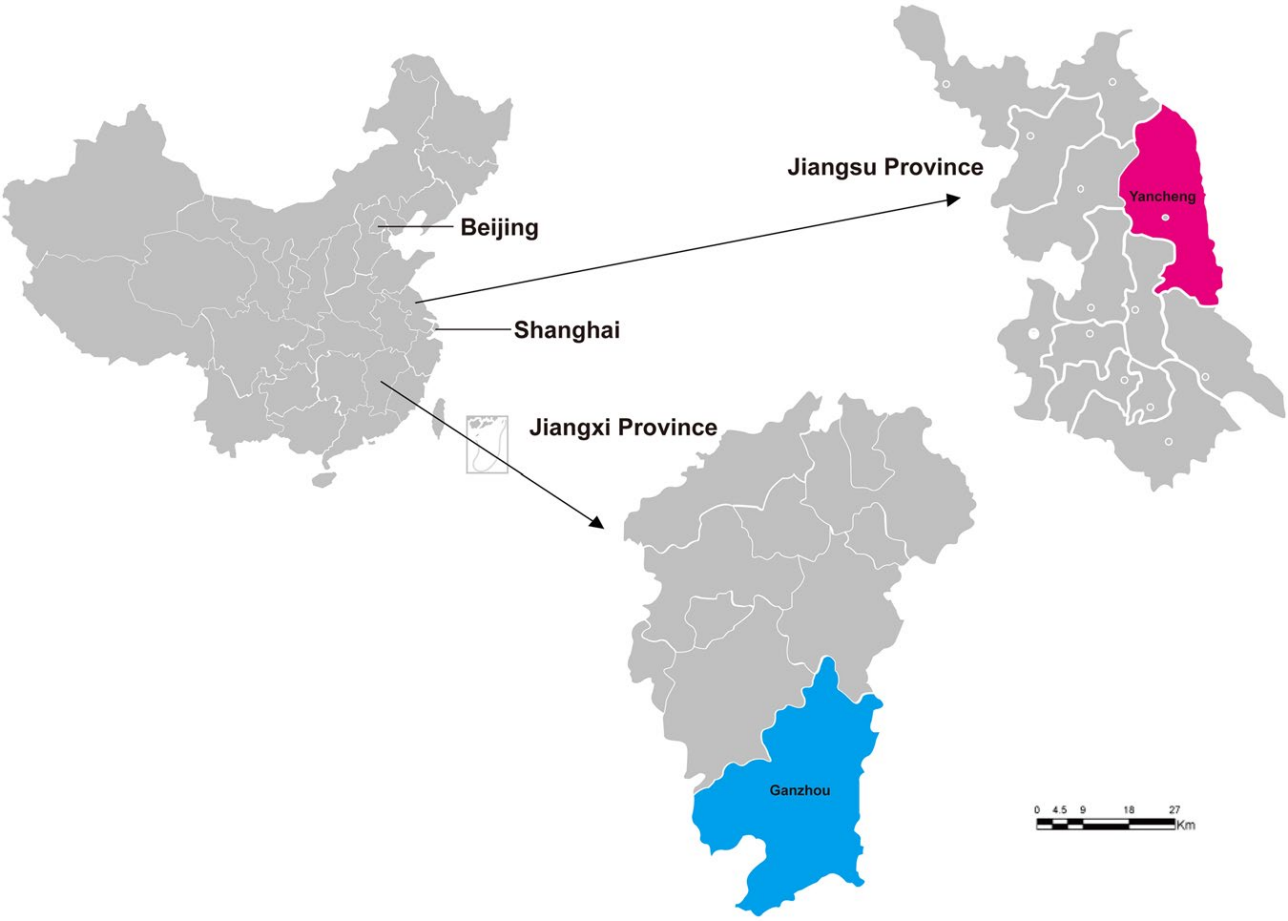
Map showing the location of sample collection sites in Yancheng city (Jiangsu province) and Ganzhou city (Jiangxi province), China.

Between 2013–2015, 72 and 62 blood samples were collected from patients with suspected Rickettsiales infection from two hospitals in Ganzhou and Yancheng cities, respectively (Fig. 2). Of these, 61.19% (82/134) were male and 38.81% (56/134) were female, with an age range from 12–88 years. All the patients had fever, and some had a series of other symptoms, including headache (80%), dizziness (74%), myalgia (85%), rash (72%), eschar (70%), and lymphadenopathy (65%).

### Rickettsiales bacteria identified in patients

Blood samples were collected at day 1 of admission (3–8 days post the onset of fever). Specific IgG and IgM antibodies against spotted fever group Rickettsiae (SFGR), *Anaplasma phagocytophilum*, *O*. *tsutsugamushi*, *Ehrlichia chaffeensis*, *R*. *prowazekii*, and *R*. *typhi* in sera were detected for each of 134 patients by IFA assays. As described in Table 1, two patients from Jiangsu were strongly positive for SFGR and *O*. *tsutsugamushi*, respectively, while one patient from Jiangxi was positive for *A*. *phagocytophilum*. However, the levels of specific IgG and IgM antibody against these three agents were relatively lower in remaining patients than three positive patients. Finally, antibody tests against *E*. *chaffeensis*, *R*. *prowazekii*, and *R*. *typhi* were all negative. Combined, these data suggest that the three patients might be infected with SFGR, *O*. *tsutsugamushi* and *A*. *phagocytophilum*.

**Table 1:** Serologic analysis of samples from three patients by indirect IFA, Jiangsu and Jiangxi provinces, China, 2013–2015.

Pathogen		Jiangsu			Jiangxi	
		Patient JSHMFN-77	Patient JSHFFN-74	Other 60 patients	Patient JXHFRJ-71	Other 71 patients
SFGR^*^	IgG titers	1:2048	—	1:64 (11/60)	—	1:64 (13/71)
	IgM titers	1:128	—	—	—	—
*A*. *phagocytophilum*	IgG titers	—	—	1:64 (2/60)	1:1024	1:64 (5/71)
	IgM titers	—	—	—	1:80	—
*O*. *tsutsugamushi*	IgG titers	—	1:1024	1:64 (16/60)	—	1:64 (15/71)
	IgM titers	—	1:64	—	—	—

^*^SFGR: Spotted Fever Group Rickettsiae.

To better determine the etiologic agents in these 134 patients, DNA extracted from their blood were screened for both the 16S ribosomal RNA *(rrs)* and 56 kDa type-specific antigen (TSA56) genes. Consequently, the *rrs* gene was recovered from two patients - JSHMFN-77 and JXHFRJ-71 - while the TSA56 genes was obtained from the patient JSHFFN-74. However, our attempts to amplify either the *rrs* or the TSA56 gene from the remaining patients failed. Genetic analysis (blastn with Nucleotide collection nr/nt) of the recovered *rrs* and TSA56 gene sequences revealed that they were most closely related to those of *R*. *monacensis* (SFGR) (99.41%), *A*. *bovis* (98.75%), and *O*. *tsutsugamushi* (99.44%), respectively (see Supplementary Table S1). Hence, these data indicate that three patients were infected with *R*. *monacensis*, *A*. *bovis*, and *O*. *tsutsugamushi*. In addition, these three patients also displayed the clinical features typical of their corresponding disease (Table 2). In sum, both the PCR and IFA tests confirmed that these patients were indeed infected with the Rickettsiales bacteria recently or previously, while their recent bacterial infection was demonstrated through the appearance of the typical clinical features.

**Table 2:** Epidemiological and clinical characteristics of patients with Rickettsiales infection in Jiangsu and Jiangxi provinces, China, 2013–2015.

Characteristic	Patient No.		
	Patient JXHFRJ-71	Patient JSHFFN-74	Patient JSHMFN-77
Age	69	65	83
Sex	F	F	M
Wildlife contact	Yes	Yes	Yes
Fever	Yes	Yes	Yes
Highest temperature, °C	39.0	38.8	39.0
Rigor	Yes	Yes	Yes
Headache	Yes	No	Yes
Dizziness	No	No	Yes
Myalgia	Yes	Yes	Yes
Rash	Yes	Yes	Yes
Eschar	Yes (5)	No	No
Lymphadenopathy	Yes	Yes	No

### Rickettsiales bacteria identified in rodents, shrews and ticks

Rodents, shrews and ticks were concurrently collected from Yancheng and Ganzhou cities. Overall, a total of 130 mice (128 striped field mice (*Apodemus agrarius*) and two house mice (*Mus musculus*) were captured from Yancheng (Table 3). *Rickettsia* bacteria (*R*. *heilongjiangensis*, *R*. *japonica*, and Uncultured *Rickettsia* like bacteria) and *O*. *tsutsugamushi* were identified in these rodents, with an overall prevalence of 9.23%. In addition, 43 rodents including striped field mice (*A*. *agrarius*) and lesser ricefield rats (*Rattus losea*) and 43 Asian house shrews (*Suncus murinus*) were captured in Ganzhou. *Rickettsia parkeri-like* strain, *R*. *raoultii*, *A*. *phagocytophilum*, *Ehrlichia* sp. and *“Candidatus* Neoehrlichia mikurensis” were identified in these rodents and shrews, with an overall prevalence of 13.95%.

**Table 3:** Prevalence of Rickettsiales bacteria in small mammals and ticks in Jiangxi and Jiangsu provinces, China.

Rickettsiales bacteria species		Jiangsu			Jiangxi			
		*Apodemus agrarius*	*Mus musculus*	*Haemaphysalis longicornis*	*A*. *agrarius*	*Rattus losea*	*Suncus murinus*	*Rhipicephalus microplus*
*Rickettsia*	*R*. *heilongjiangensis*	2/128#	0/2	0/213	0/10	0/33	0/43	0/145
	*R*. *japonica*	2/128	0/2	39/213	0/10	0/33	0/43	1/145
	*Rickettsia* like bacteria	3/128	1/2	0/213	0/10	0/33	0/43	0/145
	*Rickettsia parkeri-like*	0/128	0/2	0/213	0/10	1/33	0/43	0/145
	*R*. *raoultii*	0/128	0/2	0/213	0/10	1/33	0/43	0/145
*Orientia*	*O*. *tsutsugamushi*	4/128	0/2	0/213	0/10	0/33	0/43	0/145
*Ehrlichia*	*Ca*. N. mikurensis^*^	0/128	0/2	0/213	1/10	3/33	1/43	0/145
	*E*. *chaffeensis*	0/128	0/2	0/213	0/10	1/33	0/43	0/145
	*Ehrlichia* sp.	0/128	0/2	0/213	0/10	1/33	0/43	0/145
*Anaplasma*	*A*. *phagocytophilum*	0/128	0/2	0/213	2/10	1/33	0/43	0/145
Total		11/128	1/2	39/213	3/10	8/33	1/43	1/145

^*^Ca. N. mikurensis: *Candidatus* Neoehrlichia mikurensis.

^#^PCR positive/Samples collected.

Finally, ticks were also collected from both Yancheng and Ganzhou cites: 213 adult *Haemaphysalis longicornis* ticks (117 male, 96 female) were sampled from Yancheng, while 145 adult *Rhipicephalus microplus* ticks (65 male, 80 female) were collected in Ganzhou. Interestingly, only *R*. *japonica* was identified in these ticks sampled from both regions.

### Phylogenetic analysis of Rickettsiales

**Figure 3:**
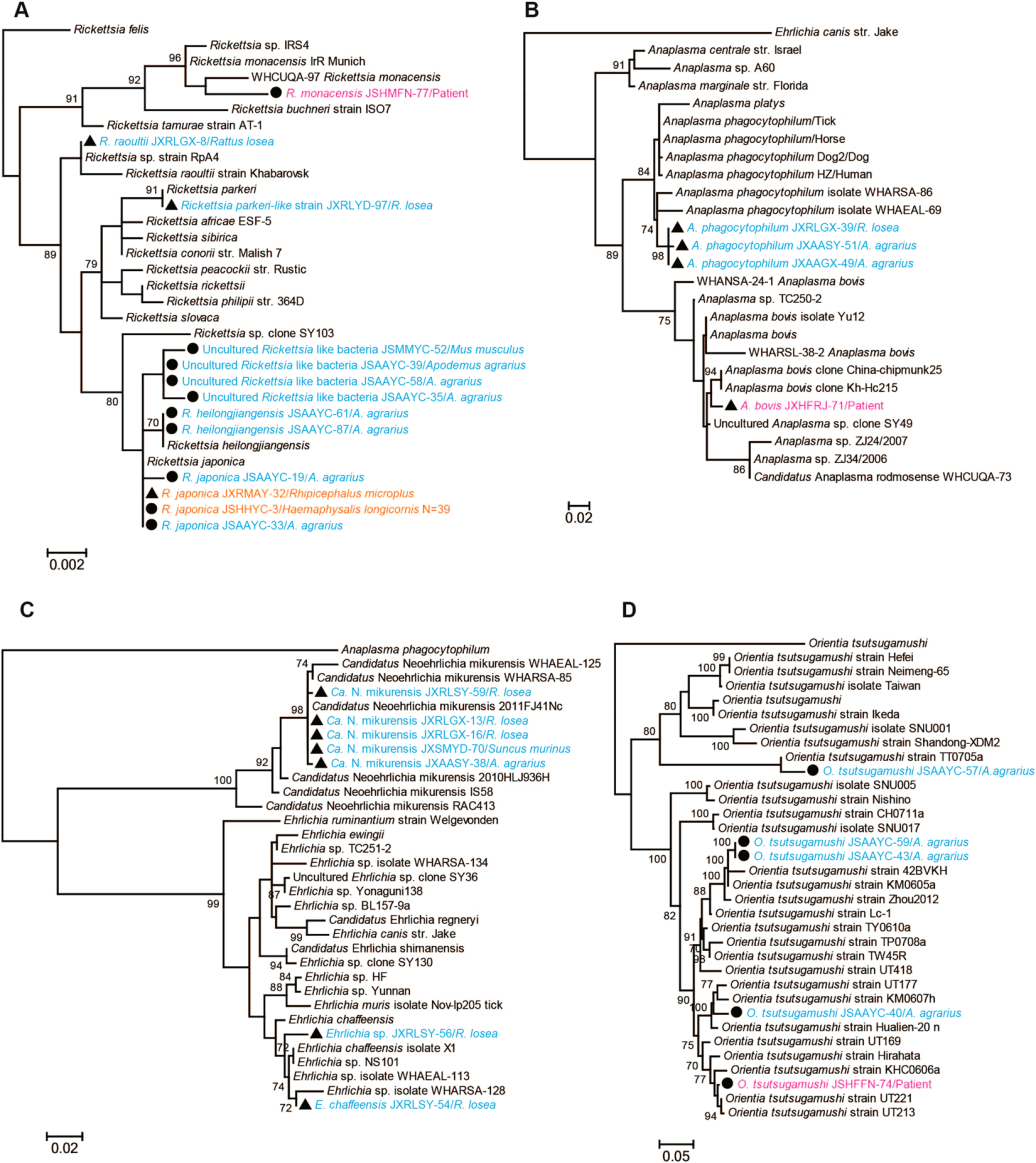
Phylogenetic trees based on the partial *rrs* gene sequences of *Rickettsia* (**A**), *Anaplasma* (**B**), *Ehrlichia* (**C**), and the TSA56 gene sequences of *Orientia tsutsugamushi* (**D**). All trees were mid-point rooted for clarity only. Bootstrap values (>70%) are shown for appropriate nodes. The scale bar represents number of nucleotide substitutions per site. The sequences from patient samples are marked in red, those from rodents and shrews are marked in blue, and sequences from ticks are marked in orange.

Phylogenetic analysis of the recovered bacterial sequences revealed a diverse array of Rickettsiales bacteria in Yancheng and Ganzhou. In the *rrs* tree (Fig. 3A), six determinded or candidatus species of *Rickettsia* (*R*. *heilongjiangensis*, *R*. *japonica*, *R*. *monacensis*, *Rickettsia parkeri-like* strain, *R*. *raoultii*, and Uncultured *Rickettsia* like bacteria) could be defined. Notably, the sequence *R*. *monacensis* JSHMFN-77/Patient recovered from the patient in Yancheng was closely related to *R*. *monacensis* WHCUQA-97 previously identified in *Culex quinquefasciatus* mosquito from Wuhan (Hubei province, China)^24^. The sequences Uncultured *Rickettsia* like bacteria JSMMYC-52/*M*. *musculus*, Uncultured *Rickettsia* like bacteria JSAAYC-39/*A*. *agrarius*, Uncultured *Rickettsia* like bacteria JSAAYC-58/*A*. *agrarius*, and Uncultured *Rickettsia* like bacteria JSAAYC-35/*A*. *agrarius* recovered from mice in Yancheng formed a distinct lineage and were most closely related to *R*. *heilongjiangensis* and *R*. *japonica*. Additionally, the sequences *R*. *raoultii* JXRLGX-8/*R*. *losea* and *Rickettsia parkeri-like* strain JXRLYD-97/*R*. *losea* identified in lesser ricefield rats from Ganzhou were closely related to *R*. *raoultii* and *R*. *parkeri*, respectively. Finally, the remaining sequences, including one sampled from *R*. *microplus* in Ganzhou, were closely related to *R*. *heilongjiangensis* and *R*. *japonica*. Hence, these data clearly indicate the co-circulation of multiple human rickettsial pathogens in both two geographic regions. The sequences of the *groEL* gene were recovered from some rodents and ticks, and their position in the *groEL* phylogeny was consistent with that of the *rrs* tree (see Supplementary Fig. S1).

**Figure 4:**
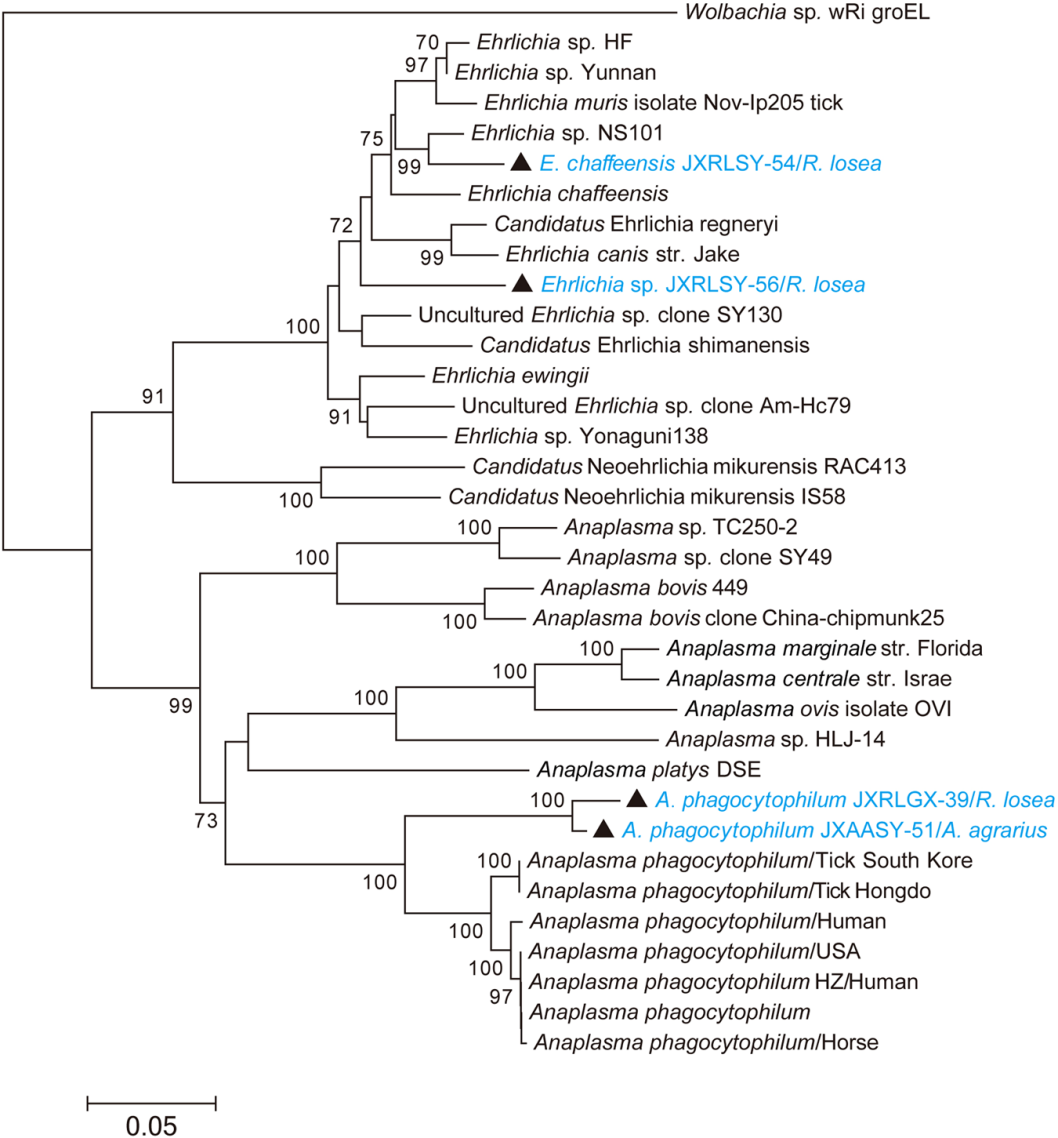
Phylogenetic tree based on partial *groEL* gene sequences of the Anaplasmataceae. The tree was mid-point rooted for clarity only. Bootstrap values (>70%) are shown for appropriate nodes. The scale bar represents number of nucleotide substitutions per site. Taxa shown in blue were obtained from rodents and shrews.

In the TSA56 gene phylogeny, *O*. *tsutsugamushi* sequences sampled from the striped field mice in Yancheng fell into two lineages (Fig. 3D). Sequence *O*. *tsutsugamushi* JSAAYC-57/*A*. *agrarius* was closely related to *O*. *tsutsugamushi* strain TT0705a isolated from a patient in Taiwan^25^, while the remaining sequences clustered together with those sampled from patients and animals in Taiwan and South East Asia^25,26^. Notably, both *A*. *bovis* and *A*. *phagocytophilum* were identified in Ganzhou city. More importantly, *A*. *bovis* sampled from one patient in Ganzhou was closely related to *A*. *bovis* clone China-chipmunk25 and *A*. *bovis* clone Kh-Hc215, identified in the common chipmunk (JX092096.1) and ticks (JX092094.1), respectively. Hence, these bacteria could cause human disease. Interestingly, *“Candidatus* Neoehrlichia mikurensis” was identified in mice, rats and shrews, and was closely related to *“Ca*. N. mikurensis 2011FJ41Nc” previously identified in *Niviventer confucianus* from Fujian province (JQ359046.1). Finally, *Ehrlichia* sequences were also recovered from lesser ricefield rats and exhibited a close evolutionary relationship to *E*. *chaffeensis* in the *rrs* gene tree. However, the sequence *Ehrlichia* sp. JXRLSY-56/*R*. *losea* formed a distinct lineage in the *rrs* and *groEL* gene trees, suggesting that they may represent a new species of *Ehrlichia* in mice (Figs 3C and 4).

## Discussion

Diseases caused by Rickettsiales bacteria have caused both high morbidity and mortality in China^27^. Although murine typhus and epidemic typhus are considered to be controlled, at least 929 cases have been registered annually since 1999 (range 929 to 6149 cases) (unpublished data obtained from Infectious Disease Report Information Management system of China Center for Diseases Control and Prevention (CDC)). Scrub typhus initially emerged in northern China and then re-emerged in southern China^13,16,28^. In addition, although HGA and HME cases are commonly reported in Chinese hospitals, it is not clear how many cases have occurred each year in China as a whole. The data obtained from this study and from China CDC indicated that scrub typhus has occurred in both Yancheng and Ganzhou cities (Fig. 1), and that other diseases caused by rickettsial pathogens may be co-circulating in both localities. In addition, as we only sampled a single hospital in each region, the true number of human cases is likely to be far greater than that recorded. Thus, there is clearly an urgent need to establish a national surveillance system in China and strengthen the laboratory diagnostic in hospitals.

Currently, the genus *Anaplasma* contains six recognized bacterial species^8^. Of these, *A*. *marginale*, *A*. *centrale* and *A*. *ovis* are known to be ruminant-specific pathogens, while *A*. *platys* is a causative agent of infectious cyclic thrombocytopenia in dogs and cats^29^. In addition, *A*. *bovis* is thought to be the agent of bovine ehrlichiosis, which frequently occurs in Africa and Asia^30,31^ and is characterized by fluctuating fever lymphadenopathy, depression, and occasionally death^32^. To date, only *A*. *phagocytophilum* (genus *Anaplasma*) is known to cause disease in humans (and sheep). Notably, however, we identified *A*. *phagocytophilum* in both mice and rats sampled from Ganzhou (Jiangxi province) and, remarkably, an *A*. *bovis* sequence was recovered from one patient exhibiting fever (up to 39 °C), rigor, headache, myalgia, rash, eschar, and lymphadenopathy. Our data therefore suggest that *A*. *bovis* may be the etiologic agent of human infection. Additional studies are clearly needed to determine whether this bacterium indeed causes disease in both humans and animals as in the case of *A*. *phagocytophilum*.

Spotted fever is a type of tick-borne disease caused by members of the SFGR. Several agents including *R*. *heilongjiangensis*, *R*. *sibirica*, *R*. *raoultii* and *Candidatus R*. *tarasevichiae* are known to cause this disease in China^12,33–35^. *R*. *japonica* harbored by multiple species of tick is also thought to cause spotted fever disease in Japan^36^, while *R*. *monacensis* is considered an agent of spotted fever-like disease in Europe^1,37–39^. Recent studies have shown that SFGR bacteria are widespread in China^40,41^. We identified *R*. *heilongjiangensis*, *R*. *japonica*, *R*. *raoultii* and Uncultured *Rickettsia* like bacteria in rodents and ticks from Yancheng and Ganzhou. As such, these data indicate the co-circulation of multiple members of SFGR in rodents and ticks in both two regions. More importantly, *R*. *monacensis* was recovered from one spotted fever patient in Yancheng, implying that the infection might be caused by this bacterium. As spotted fever-like diseases commonly observed in Chinese hospitals, especially during spring and summer, further studies are needed to determine the prevalence of SFGR bacteria in humans and animal reservoirs.

Although scrub typhus occurs only in Asia, it is estimated that more than one million cases of the disease are transmitted annually with more than one billion people at risk^13–16,42^. Scrub typhus has a long history in China, leading to high morbidity and mortality in humans^27^, particularly in southern China^13,14,28^. Although chiggers (family *Trombiculidae*) are thought to be the true reservoir of *O*. *tsutsugamushi*, rodents including mice and rats also play an important role in the transmission of *Orientia* bacteria^43,44^. Even though it is the only recognized species within the genus *Orientia*, *O*. *tsutsugamushi* exhibits high genetic diversity^45–47^. We identified *O*. *tsutsugamushi* in one patient as well as the striped field mice sampled from Yancheng. Notably, the recovered bacterial sequence was phylogenetically diverse (Fig. 3D), and that recovered from the patient did not cluster with those from local mice. Hence, these data suggest the co-circulation of multiple genotypes of *O*. *tsutsugamushi* in Yancheng.

In conclusion, we have shown that scrub typhus is present in Yancheng city, Jiangsu province, China, while other Rickettsiales pathogens are co-circulating in both Jiangsu and Jiangxi provinces. In addition, we documented multiple species of Rickettsiales bacteria in rodents, shrews, and ticks, indicating a considerable natural diversity of Rickettsiales bacteria in both geographic regions. Hence, our data highlight the urgent need for the regular surveillance of local arthropods, mammals and humans for evidence of Rickettsiales infection in China.

## Materials and Methods

### Sample collection

A total of 62 blood samples were collected from patients experiencing fever at Funing People’s Hospital, Yancheng city, Jiangsu province, during May 2013 to November 2015. Similarly, 72 blood samples were collected from patients presenting with fever at the First Affiliate Hospital of Gannan Medical University, Ganzhou city, Jiangxi province, China, during May 2013 to November 2015 (Fig. 2). Patients who had fever (>37 °C) and clinical symptoms including rash and eschar, as well as a history of wildlife contact, were enrolled and screened for infection with Rickettsiales bacteria. Patients with another obvious cause of fever (e.g., pneumonia, cellulitis, etc.), were excluded. Four ml whole blood with ethylenediamine tetraacetic acid dipotassium salt dihydrate (EDTA-2K) and two ml sera were collected from each of 134 patients. Information such as the date of the onset of illness, fever, and clinical symptoms was obtained from the relevant hospitals.

Small mammals and ticks were also collected in Yancheng and Ganzhou cities during April 2015 to October 2017. Rodents and shrews were captured with snap-traps, set at 5 meters intervals and baited with deep-fried dough sticks in both two cities. Most of ticks were directly picked from infested wild and domestic animals, although a few were collected using a tick drag-flag method. In addition, records of scrub typhus cases were obtained from the China Center for Diseases Control and Prevention.

### Ethical approval

Signed individual written informed consent was obtained from each patient at the time of sample collection. All adult human subjects provided informed consent, and a parent or guardian of any child participant provided informed consent on the child’s behalf (less than 18 years old). Collecting human serum samples from patients was reviewed and approved by the ethics committees of the National Institute for Communicable Disease Control and Prevention, China CDC. All methods were performed in accordance with the relevant guidelines and regulations. Animal experiments were performed according to *Guidance for Experimental Animal Welfare and Ethical Treatment* by the Ministry of Science and Technology of China (www.most.gov.cn/fggw/zfwj/zfwj2006/zf06yw/zf06qt/200612/t20061226_39235.htm). These protocols were approved by the National Institute for Communicable Disease Control and Prevention of the China CDC (Permit number: ICDC20170616-001). All mammals were euthanized used the ether anesthetic chamber, and all tissue samples were taken during necropsy. Tissue samples of liver, spleen, lung, and kidney were collected from small mammals for detecting Rickettsiales bacteria. Field Research was also approved by the ethics committee of National Institute of Communicable Disease Control and Prevention of the China CDC, and the field permit number is 201710082.

### Serological assays

Serum samples were screened by indirect immunofluorescence assay (IFA) for IgG and IgM against spotted fever group Rickettsiae (SFGR), *A*. *phagocytophilum*, *O*. *tsutsugamushi*, *Ehrlichia chaffeensis*, *R*. *prowazekii*, and *R*. *typhi* by using IFA kit (Focus and Fuller, USA). As indicated in the manufacturer’s instruction, IgG titers ≥64 and IgM titers ≥20–64 were considered as indicating a positive result.

### DNA extraction and PCR assays

According to the manufacturer’s instructions, 200 μl human whole blood was used to extract DNA with the QIAamp DNA Blood Mini Kit (QIAGEN, Germany). Nested or semi-nested PCRs targeting the 16S ribosomal RNA (*rrs*) and 56 kDa type-specific antigen (TSA56) genes was performed to detect Rickettsiales bacteria as described previously^15,19^. The 1400 bp *rrs* gene was amplified using the primers Eh-out1/Eh-out2 (outer primers) and Eh-gs1/Eh-gs2 (inner primers), while the 600 bp TSA56 gene was amplified using the primers Otr56_498F/r56_2057 (outer primers) and r56_585F/r56_2057 (inner primers). Amplified positive DNAs were purified and sequenced in both directions (Sangon, Shanghai, China).

DNA was extracted individually from all ticks using the QIAamp DNA Mini Kit (Qiagen, Germany) according to the manufacturer’s instructions, and from spleen and liver tissues of small mammals using QIAamp DNeasy Blood & Tissue Kit (Qiagen, Germany) according to the manufacturer’s instructions. The spleen and liver from small mammals were homogenized. Tissue suspension (400 μl) was used to extract DNA. Individual ticks were ground with 500 μl or 1 ml PBS using pestl, and the whole suspension was used to extract DNA. Rickettsiales DNA was detected using nested or semi-nested PCR targeting the *rrs* (length of 1400 bp), *groEL* (length of 1300 bp), and TSA56 (length of 900 bp) genes as described previously^15,19,24^, and fragments of the expected size were purified and sequenced in both directions (Sangon, Shanghai, China).

### Sequence data and phylogenetic analyses

DNA sequences of the *rrs*, *groEL*, and TSA56 genes recovered from positive samples were aligned with existing reference sequences taken from GenBank using the ClustalW protocol (default parameters) as implemented in the MEGA program, version 6.06^48^. The bacterial sequences obtained were named according to their species, geographic origins, hosts, and sample numbers. Phylogenetic trees of the data were estimated using the Maximum Likelihood (ML) method employing the GTR + Γ + I model of nucleotide substitution as implemented in PhyML (version 3)^49^. 1000 bootstrap replicates were generated to determine the level of support for individual nodes on the trees, and all trees were mid-point rooted for purposes of clarity. All sequences generated here have been submitted to GenBank and assigned accession numbers MH722222 to MH722254.

## Supplementary information

Supplementary Information
