# Supplementary material for: Epidemiology and Diversity of Rickettsiales Bacteria in Humans and Animals in Jiangsu and Jiangxi provinces, China
Source: Sci Rep. 2019 Sep 11;9:13176. doi: 10.1038/s41598-019-49059-3 (PMC6739303; doi:10.1038/s41598-019-49059-3)
Supplement: Supplementary file 1 — Supplementary Information [file 41598_2019_49059_MOESM1_ESM.pdf]

Epidemiology and Diversity of Rickettsiales Bacteria in Humans and Animals in Jiangsu and Jiangxi provinces, China

Running title: Infection of Rickettsiales bacterial in China

Miao Lu[a1], Feng Li[b1], Yong Liao[c1], Jin-Jin Shen[b], Jian-Min Xu[d], Yin-Zhong Chen[b], Jian-Hua Li[c], Edward C. Holmes[aef], Yong-Zhen Zhang[af2]

[a] State Key Laboratory for Infectious Disease Prevention and Control, Collaborative Innovation Center for Diagnosis and Treatment of Infectious Diseases, National Institute for Communicable Disease Control and Prevention, Chinese Center for Disease Control and Prevention, Changping, Beijing, China

[b] Yancheng Center for Disease Control and Prevention, Yancheng, Jiangsu province, China

[c] Ganzhou Center for Disease Control and Prevention, Ganzhou, Jiangxi province, China

[d] Jiangxi Center for Disease Control and Prevention, Nanchang, Jiangxi province, China

[e] Marie Bashir Institute for Infectious Diseases and Biosecurity, Charles Perkins Centre, School of Life and Environmental Sciences and Sydney Medical School, The University of Sydney, Sydney, NSW 2006, Australia.

[f] Shanghai Public Health Clinical Center & Institutes of Biomedical Sciences, Fudan University, Shanghai, China.

[1] These authors contributed equally to this article.

[2] Correspondence to: Dr. Yong-Zhen Zhang, State Key Laboratory of Infectious Disease Prevention and Control, National Institute of Communicable Disease Control and Prevention, Chinese Center for Disease Control and Prevention, Changping Liuzi 5, Beijing, 102206, China.

Tel: 086-10-58900782; Email: zhangyongzhen@icdc.cn.

Supplementary Table S1. Bacterial sequences obtained in this study.

Strains	Genes(nt)			Bacteria[a]
	rrs	*groEL*	*TSA56*	
**Jiangsu Province**				
**Human**				
JSHMFN-77	1356 (99.41%)[b]	-[c]	-	*R. monacensis*
JSHFFN-74	-	-	540 (99.44%)	*O. tsutsugamushi*
Apodemus agrarius				
JSAAYC-40	-	-	738 (99.86%)	*O. tsutsugamushi*
JSAAYC-43	-	-	708 (99.86%)	*O. tsutsugamushi*
JSAAYC-57	-	-	882 (98.60%)	*O. tsutsugamushi*
JSAAYC-59	-	-	711 (99.86%)	*O. tsutsugamushi*
JSAAYC-61	1091 (100.0%)	-	-	*R. heilongjiangensis*
JSAAYC-87	1091 (100.0%)	-	-	*R. heilongjiangensis*
JSAAYC-19	795 (99.87%)	-	-	*R. japonica*
JSAAYC-33	790 (100.0%)	807 (100.0%)	-	*R. japonica*
JSAAYC-35	910 (99.89%)	663 (99.50%)	-	*R. japonica*
JSAAYC-39	910 (99.89%)	-	-	*R. japonica*
JSAAYC-58	910 (99.89%)	-	-	*R. japonica*
Mus musculus				
JSMMYC-52	910 (99.89%)	-	-	*R. japonica*
Haemaphysalis longicornis				
JSHHYC-3 (N=39)	1303 (100%)	702 (99.75%)	-	*R. japonica*
**Jiangxi Province**				
**Human**				
JXHFRJ-71	957 (98.75%)	-	-	*A. bovis*
A. agrarius				
JXAASY-38	1376 (99.78%)	-	-	*Ca.* N. mikurensis
JXAAGX-49	1316 (99.70%)	-	-	*A. phagocytophilum*
JXAASY-51	1406 (99.57%)	1248 (92.16%)	-	*A. phagocytophilum*
Rattus losea				
JXRLYD-97	1122 (99.73%)	-	-	*R. parkeri*
JXRLGX-8	895 (100.0%)	750 (100.0%)	-	*R. raoultii*
JXRLGX-13	1376 (100.0%)	-	-	*Ca.* N. mikurensis
JXRLGX-16	1376 (100.0%)	-	-	*Ca.* N. mikurensis
JXRLSY-59	1384 (99.93%)	-	-	*Ca.* N. mikurensis
JXRLSY-54	717 (99.72%)	1000 (94.44%)	-	*E. chaffeensis*
JXRLSY-56	717 (99.30%)	1003 (91.75%)	-	*E. chaffeensis*
JXRLGX-39	1406 (99.72%)	1248 (91.74%)	-	*A. phagocytophilum*
Suncus murinus				
JXSMYD-70	1376 (100.0%)	-	-	*Ca.* N. mikurensis
Rhipicephalus microplus				
JXRMAY-32	1337 (100.0%)	801 (99.75%)	-	*R. japonica*

[a] The abbreviation is the same as used in Table3.

[b] The length of sequence amplified from the samples (Nucleotide sequence identity compared to the reference sequences from GenBank).

[c] “-”, not available.

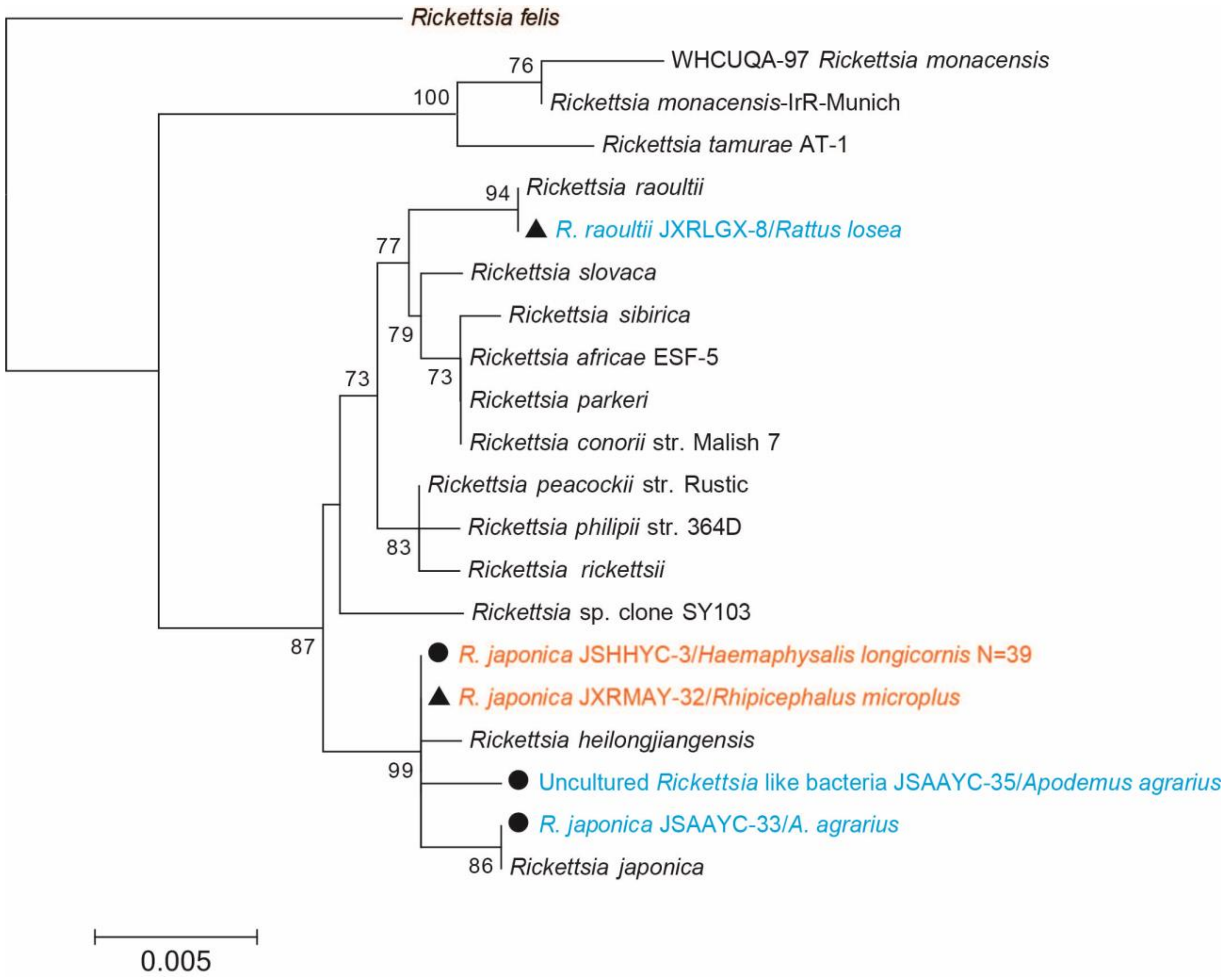

Supplementary Figure S1: Phylogenetic tree based on partial *groEL* gene sequences of *Rickettsia.* The tree was mid-point rooted for clarity only. Bootstrap values (>70%) are shown for appropriate nodes. The scale bar represents number of nucleotide substitutions per site. The sequences from rodents and shrews are marked in blue, and those from ticks are marked in orange.
